# A STING for necroptosis

**DOI:** 10.1038/s41418-025-01602-8

**Published:** 2025-10-28

**Authors:** Marlena Nastassja Schlecht, Karolin Flade, Wulf Tonnus

**Affiliations:** Medical Clinic and Policlinic 3, University Hospital CGC at the Technische Universität Dresden, Dresden, Germany

**Keywords:** Cell death and immune response, Immunological disorders

Necroptosis is a form of regulated cell death with necrotic morphology. The discovery of the small-molecule inhibitor Nec-1 and its target, RIPK1 (*Receptor interacting protein kinase 1*), established a paradigm shift by demonstrating that regulated cell death extends beyond apoptosis [1]. Three major induction pathways have since been defined: TRIF-dependent necroptosis downstream of TLR3/7, ZBP1-mediated (*Z-RNA binding protein 1*) necroptosis upon intracellular Z-RNA-detection, and the canonical TNFR1–RIPK1 axis, the latter being the best characterized. A complex regulatory network controlling the delicate balance of extrinsic apoptosis and necroptosis has been elucidated, placing activity of Casp8 (*Caspase 8*) in the center of the necroptosis-suppressing machinery. Stimulation of TNFR1 (*Tumor necrosis factor receptor 1*) leads to phosphorylation of RIPK1, which relieves steric inhibition of ZBP1-mediated RIPK3 (*Receptor interacting protein kinase 3*) oligomerization upon loss of Casp8 function, culminating in necroptosis [2]. Genetic ablation of Casp8 leads to prenatal lethality, which can be rescued by loss of RIPK3. Notably, loss of TNFR1 does not fully rescue Casp8-deficiency; instead, it precipitates multi-organ inflammation with prominent dermatitis, a phenotype shared with deficiencies in other necroptosis-inhibiting components of the TNFR1 pathway. Dermatitis is also seen in keratinocyte-limited deletion of Casp8 (Casp8^E-ko^) or RIPK1 (RIPK1^E-ko^), respectively, underscoring that this regulatory crosstalk occurs within individual epithelial cells rather than being solely mediated by infiltrating immune cells. Nevertheless, the identity of the non-TNFR1-dependent factor that drives necroptosis in the absence of Casp8 has remained unresolved. In the current volume of *Nature*, the Liccardi group identified this factor as STING-mediated ZBP1 induction by an elegant series of complex murine crossing experiments [3].

Starting from evidence that dermatitis upon keratinocyte-specific loss of RIPK1 is dependent on ZBP1 [4], they confirmed ZBP1 upregulation in the skin of Casp8^−/−^/Ripk3^−/−^ and Casp8^−/−^/Mlkl^−/−^ mice, even in the absence of dermatitis. To investigate the source of a putative type I interferon signal upregulating ZBP1, they performed bulk sequencing on skin samples of newborn (P5) Casp8^E-ko^ mice and found, next to the expected upregulation of interferon-stimulated genes such as *Zbp1* and *Mlkl*, a strong correlation to the upregulated STING (*Stimulator of interferon genes*) signaling pathway. Unexpectedly, immunostainings demonstrated STING upregulation in keratinocytes rather than infiltrating CD45+ immune cells, pointing to a cell-intrinsic mechanism priming them to necroptosis. In MEFs, a STING agonist induced ZBP1 expression, which could be blocked by an antagonist or genetic deletion of STING. Furthermore, STING agonism primed for TNFα-induced necroptosis and induced necroptosis upon Casp8-deficiency – both effects being dependent on ZBP1. Whereas TNFa-induced necroptosis involves formation of a FADD-RIPK1-RIPK3 complex (canonical necrosome), STING activations was found to induce a ZBP1-RIPK1-RIPK3 complex. Compared to Casp8^E-ko^/Tnfr1^−/−^ mice, Casp8^E-ko^/ Sting^E-ko^ mice demonstrated a small but significant survival advantage (P10 vs P11), and featured partial rescue of lethal dermatitis. Importantly, Casp8^E-ko^/Tnfr1^−/−^/Sting^E-ko^ mice showed only late signs of dermatitis and multiorgan inflammation at 14 weeks of age, whereas Casp8^E-ko^/Tnfr1^−/−^/Zbp1^−/−^ mice were completely rescued throughout adulthood. As this suggested non-keratinocyte-restricted effects of STING, the authors compared Casp8^E-ko^/Tnfr1^−/−^ to Casp8^E-ko^/Sting^−/−^ mice, with the latter demonstrating longer survival and attenuated dermatitis. Contrasting Casp8^E-ko^/Tnfr1^−/−^/Sting^E-ko^ mice, Casp8^E-ko^/Tnfr1^−/−^/Sting^−/−^ mice were fully rescued (alike to Casp8^E-ko^/Tnfr1^−/−^/Zbp1^−/−^ mice). Importantly, Casp8^E-ko^/Zbp1^−/−^ mice phenocopied the severe phenotype of Casp8^E-ko^ mice. These findings also underscore the complex interactions of cell death and immune cell dynamics (“necroinflammation”) [5].

**Fig. 1:**
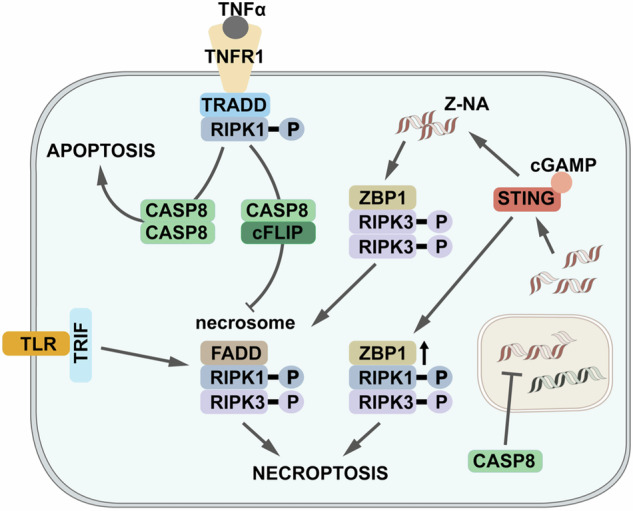
STING as novel checkpoint on necroptosis induction. Different pathways lead to necroptosis, with the TNFR1-dependent one being the best characterized one leading to formation of “necrosome” complex consisting of FADD-RIPK1-RIPK3, whose formation is blocked by Caspase 8 activity. Additionally, Z-handed DNAs and RNAs (“Z-NAs”) activate ZBP1 leading to formation of an alternative necrosome consisting of ZBP1-RIPK1-RIPK3. This is promoted by i) STING-mediated ZBP1 upregulation and ii) accumulation of nucleotides upon Caspase 8-deficiency.

Taken together, these findings established STING-induced ZBP1-expression as a novel and TNFR1-independent checkpoint of necroptosis induction (Fig. 1), contributing to the mechanistic framework of previous reports on ZBP1-induced necroptosis [4, 6, 7]. Yet, the factor activating STING remained to be elucidated. It those terms, the authors elegantly excluded a role of mitochondrial DNA (a typical activator of cGAS/STING), but rather found nuclear DNA leakage by Casp8-dependent compromises in genome integrity to be responsible. This fits to previous reports on ZBP1 being involved in cell death upon replicative crisis [8]. Additionally, STING promoted ZBP1 activation by fostering its increased exposure to Z-RNAs, complementing previous reports on ZBP1 stabilizing Z-DNAs to support cGAS/STING activation [9] – this mutual feedback-loops highlighting the close relationship of these factors.

But why are these complex studies in Casp8-deficient conditions important? How does this relate to human pathophysiology? The authors offer a glimpse to these questions by investigating the role of necroptosis in SAVI syndrome (*STING-associated vasculopathy with onset in infancy*)—an ultra-rare Mendelian disease caused by mutations permitting constitutive STING activation, for which current treatments interfering with interferon signaling only yield partial symptom control. In a cohort of children with SAVI, they observed profound upregulation of cytokines involving type I interferons, and a corresponding priming towards necroptosis indicated by upregulation of mRNA for *RIPK3*, *MLKL*, and *ZBP1*. These signatures demonstrated significant overlap with profiles of Casp8^E-ko^ mice. As a proof of principle, they turned to mice harboring an activating STING mutation recapitulating features of human disease. These mice demonstrated necroptosis induction throughout various tissues, and co-deletion of RIPK3 completely abolished their inflammatory phenotype. In line with the mechanistic concept proposed here, it was recently demonstrated that deletion of TNFR1 only provides partial rescue in this model [10]. As inhibitors of necroptosis are at an advanced stage of clinical trials, these findings yield hope to patients with SAVI for future therapies.

The implications of these findings, however, extend well beyond an ultra-rare orphan disease. Blistering lesions, for example, are common inflammatory manifestations with features distinct from apoptosis. Moreover, several viruses encode caspase inhibitors [11], echoing aspects of the Casp8-deficient mouse model. Such viruses continue to cause substantial morbidity and mortality in immunocompromised patients, with treatment currently limited to virostatics. A similar paradigm applies to influenza, where ZBP1-dependent cell death is a key pathogenic mechanism [12]. Collectively, the evidence presented in this study opens new avenues for innovative therapeutic strategies across a broad spectrum of infectious and inflammatory diseases.
